# Blockchain-Based Digital Contact Tracing Apps for COVID-19 Pandemic Management: Issues, Challenges, Solutions, and Future Directions

**DOI:** 10.2196/25245

**Published:** 2021-02-09

**Authors:** Sheikh Mohammad Idrees, Mariusz Nowostawski, Roshan Jameel

**Affiliations:** 1 Department of Computer Science Norwegian University of Science and Technology Gjovik Norway; 2 Department of Computer Science and Engineering Jamia Hamdard New Delhi India

**Keywords:** COVID-19, digital contact tracing, privacy preservation, security, blockchain technology, blockchain, privacy, contact tracing, app, surveillance, security

## Abstract

The COVID-19 pandemic has caused substantial global disturbance by affecting more than 42 million people (as of the end of October 2020). Since there is no medication or vaccine available, the only way to combat it is to minimize transmission. Digital contact tracing is an effective technique that can be utilized for this purpose, as it eliminates the manual contact tracing process and could help in identifying and isolating affected people. However, users are reluctant to share their location and contact details due to concerns related to the privacy and security of their personal information, which affects its implementation and extensive adoption. Blockchain technology has been applied in various domains and has been proven to be an effective approach for handling data transactions securely, which makes it an ideal choice for digital contact tracing apps. The properties of blockchain such as time stamping and immutability of data may facilitate the retrieval of accurate information on the trail of the virus in a transparent manner, while data encryption assures the integrity of the information being provided. Furthermore, the anonymity of the user’s identity alleviates some of the risks related to privacy and confidentiality concerns. In this paper, we provide readers with a detailed discussion on the digital contact tracing mechanism and outline the apps developed so far to combat the COVID-19 pandemic. Moreover, we present the possible risks, issues, and challenges associated with the available contact tracing apps and analyze how the adoption of a blockchain-based decentralized network for handling the app could provide users with privacy-preserving contact tracing without compromising performance and efficiency.

## Introduction

COVID-19, caused by the novel coronavirus SARS-CoV-2, was unknown prior to its spread in late 2019 in Wuhan, China [1]. The infection began with 27 people in Wuhan, which has now grown to more than 42 million people globally [2]. Within a month of the initial outbreak, COVID-19 was declared a global public health emergency by the World Health Organization (WHO) in January 2020. SARS-CoV-2 is usually transmitted from infected person to healthy person via physical contact [3]. Several studies also claim that the disease is highly contagious and can be transmitted through air particles, which contributes to its uncontrollable spread. Infected people can be symptomatic or asymptomatic, but both types of patients can transmit the virus. Therefore, the only way to control the spread is to keep infected individuals in isolation for 14 days, as the incubation period ranges between 1-14 days, per research carried out in Wuhan [4]. Since COVID-19 appeared suddenly, there was no information about treatment and prevention. Although clinical trials and research are being carried out globally to deal with this pandemic, the availability of a vaccine to the public may not be possible in the near future. Therefore, as of now, everybody must follow some simple practices every day like avoiding gatherings, maintaining social distance, washing hands, using hand sanitizers, and wearing masks and gloves when going out.

To combat this coronavirus, governments throughout the world are implementing several measures to avoid social contact among people to minimize spread. Complete lockdowns were imposed in several countries in which national and international borders were sealed, schools and universities were shut down, employees were asked to continue their work from home, malls and markets were closed, and gatherings and functions were suspended; all these efforts are being made to restrict human contact as much as possible. Nevertheless, the impact of these actions on the economy has raised global concerns, which makes it important to have a balance between prevention mechanisms and economic activities. Health organizations globally are keeping manual track of people who might have come into contact with COVID-19–positive persons, but this process is time consuming, prone to errors, and inefficient. Therefore, a digital contact tracing system is needed to identify, assess, and manage the people and locations that have been exposed to COVID-19–infected patients in order to prevent the spread of the virus and break the chain [5]. Digital contact tracing is a mechanism that utilizes the technological concepts as a tool to collect data to identify contacts and prevent transmission [6].

However, contact tracing means the continuous monitoring of data such as personal details and location of users and infected patients, which creates a sense of fear in society that their movements are being captured and broadcasted to the app managers. Furthermore, the privacy of individuals’ data becomes a concern in a pandemic situation, as people become unsure how the government is going to use this collected information. In this paper, an outline is provided on how containment in pandemic situations such as COVID-19 can be achieved using digital contact tracing and elaborate on the issues and challenges that this mechanism imposes on society. A brief description is also provided on the contact tracing systems implemented specifically for COVID-19, followed by a discussion on blockchain-based decentralized contact tracing, which can handle users’ data in a transparent and immutable manner, thus providing security as well as ensuring privacy.

## Digital Contact Tracing

It is necessary to have containment in order to limit the spread of communicable diseases and prevent them from reaching the level of a pandemic like COVID-19. Contact tracing is a mechanism of identifying infected people, keeping track of others who might have come in contact with them, and collecting relevant information about these contacts to take timely measures to minimize the spread of contagious diseases [6,7]. Traditional contact tracing has been employed in the past for controlling the spread of several communicable diseases. The process of traditional contact tracing is based on keeping track of people an individual has been in touch with, as well as places they have visited in the past few days or weeks. Traditionally, these details were kept on paper files, and people with a chance of infection were informed via mail or phone call. This is a tedious and error-prone method that demands too much labor. With time, everything has been digitized, including the process of contact tracing. Digital contact tracing is a mechanism that can keep track of these details through mobile phones, which can be used to identify people who might have come into contact with an infected patient [6]. The WHO has divided the mechanism of contact tracing into three steps as follows [8]:

1. Identification of contacts: when a person is diagnosed with a communicable viral disease, their contacts are identified by asking about the routine and activities of the infected person. These contacts could be anyone who had come in contact with the patient, such as family members, friends, colleagues, or health care practitioners.
2. Listing of contacts: after identifying the individuals who have come in contact with the infected person in the past few days, a list of these contacts is compiled. These contacts are then informed of the situation and guided about the practices that they must follow if they develop any symptoms. The contacts are also advised to remain under isolation or quarantine, if required.
3. Follow-up with contacts: periodic and regular follow-ups with the contacts are mandatory as they help in the timely monitoring of health symptoms and test results and prevent the further spread of the disease.

**Figure 1:**
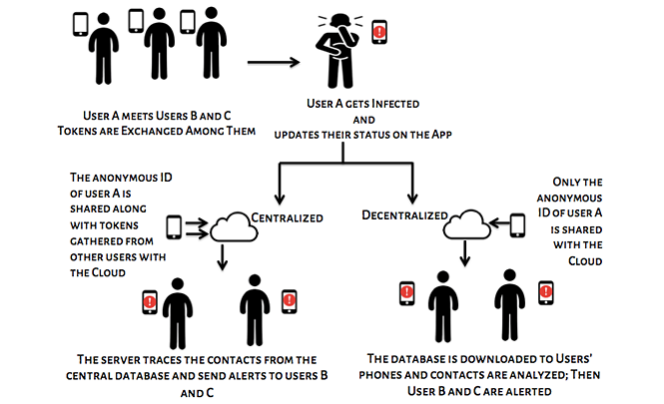
Digital contact tracing mechanism.

Nowadays, contact tracing is being implemented through smartphones. Development and deployment of several mobile apps are being done to resolve the problems associated with the traditional, manual contact tracing approaches. Bluetooth technology is one of the mainstream approaches for contact tracing apps to uncover the individuals who might have been in proximity of infected people. The Bluetooth-based approaches do not store the location or data of users; they only notify infected persons’ contacts with minimal interference to the user’s privacy. Such techniques could be centralized or decentralized. In case of decentralized techniques, the details are stored on the user’s device only, which gives users both power and control over their personal data. The analysis and processing of the data for tracking contacts are also done on the user’s device, which promotes transparency, privacy, and user consent [9]. In centralized apps, the analysis and processing are done on a central server, and users are alerted if required. Figure 1 depicts how digital contact tracing works in decentralized and centralized setups.

## Existing Digital Contact Tracing Techniques for COVID-19

There are several apps developed recently to effectively handle the COVID-19 pandemic. These apps help health care workers as well as the public to gain insight into the situation so as to make suitable decisions. Some of these apps are discussed below, followed by a comparison depicted in Table 1.

### TraceTogether

TraceTogether is an app based on BlueTrace protocols that utilizes Bluetooth low-energy technology to determine and record the proximity details of users [10]. In order to run this app, users need to keep their mobile devices in the active state, which allows for the broadcasting of their location at all times. This, however, affects battery usage. Bluetooth has always been vulnerable to security attacks (eg, sniffers), and the chances of replay attacks are also high, which might generate false information leading to panic among the public. Furthermore, concealing the hardware device associated with Bluetooth technology is difficult and can lead to the exposure of user identity. Moreover, the proximity limit of Bluetooth is limited. The TraceTogether app works on a centralized network, which means that the identities of users might not be revealed to other users, but this identity is known to a central authority and an attacker might gain control of the data by hacking the central node of the network (single point of failure).

### COVID Trace

Developed by Apple and Google, COVID Trace is an app that is based on Bluetooth technology that only collects necessary information like location with respect to time and does not retain any recognizable user data (eg, identity), thereby keeping the data anonymous [11]. In this app, if 2 individuals stay in proximity to one another for more than 10 minutes, tokens are exchanged and stored in the app. Whenever a person is diagnosed with COVID-19, this information is shared with the app server, which is referred to as escrow. These generated tokens are only shared to the public database with the permission of the user. This app keeps the users’ location records for the past 3 weeks, which is recorded only when the app user leaves their home. On the basis of the collected data, the symptoms, the area of exposure, and the number of days a person has been sick are stored. App managers are responsible for the anonymization of user location while uploading. Moreover, the exact time is not disclosed and is rounded off for privacy reasons. One of the drawbacks of this app is that the presence or absence of the disease is not verified. Nevertheless, it offers improved privacy properties as compared to storing raw Bluetooth device IDs such as in the previous example.

### HowWeFeel

This app was developed specially for researchers, health care professionals, and doctors. It collects and aggregates the symptoms and personal information of users for the purpose of sharing it with health care practitioners who are working to have a better understanding of COVID-19 and ways to combat it [12]. The collected data are utilized to better understand the nature of its spread and to identify people at risk of infection, in order to take the necessary steps on time. Data are collected for both healthy and unhealthy individuals. The app asks users certain questions in terms of how they are feeling and whether or not they have gotten a COVID-19 test, as well as the result of the test. Moreover, a unique token is issued for the identification of every user. Firewalls, antiviruses, and cryptographic algorithms are also applied to ensure authorized access.

### NOVID

NOVID is another app developed for contact tracing that utilizes the concepts of Bluetooth technology and ultrasonic sound waves for accurately counting the number of contacts to provide exact distance measurement [13]. The microphones of mobile phones are utilized in this app to listen to inaudible sounds by exploiting Bluetooth technology whenever a device passes another. Then the proximity among users is calculated without demanding any personal information or location. Whenever a new user is registered on the app, a unique user ID is generated randomly to identify the device. The user information gathered by this app does not link it with the users’ personal details such as name, email, or device without the user’s permission. The IDs of the tracked users are gathered and stored on a server, and if a person is diagnosed with COVID-19, then the user ID is encrypted and then sent to the other users of the app.

### COVID Shield

The COVID Shield app collects the IDs of individuals using Bluetooth technology in a random manner and shares it with nearby users [14]. If a person is found to be COVID-19 positive, then their ID is shared anonymously with other users with a chance of exposure to the disease. The app downloads these randomly generated IDs from the server after an interval of time, and these IDs are mapped with user information extracted from personal devices to determine one’s chances of exposure to the virus in the past few days. COVID Shield encompasses a web portal, a server, and a mobile app. No personal information is directly collected on the app; instead, a random ID is assigned along with temporary keys and logs. The app uses the Google and Apple Exposure Notification framework to maintain the privacy of users’ data. The collected data are stored on AWS (Amazon Web Services) cloud, and users have complete jurisdiction over their data. Users are able to turn their notifications off and delete their exposure log.

### Zero

The Zero app is installed on the user’s mobile device, through which data and user ID are collected and then stored on a centralized cloud storage provided by Google Cloud [9]. This app supports API (application programming interface) and TCN (Temporary Contact Numbers) protocols provided by both Apple and Google [15]. App managers can share the collected data with other entities for lawful purposes such as law enforcement or clinical research. The data remain anonymous while being shared. In order to maintain the security of the data, SSL (Secure Socket Layer) protocols are used. The users have the right to file a complaint with the app managers if their data are compromised in any way. However, the app providers do not assure the privacy of the collected information.

### ShareTrace

When a new user registers on the ShareTrace app, a random ID is generated and assigned. Personal information, such as email, name, and location, is kept safe on the user’s device, and the mobile phone of the user communicates with the app server using the assigned ID only [16]. Contact tracing is done using Bluetooth technology, which shares users’ packets (ie, Bluetooth ID of the device, symptoms, diagnosis, and contact history associated with the device) when 2 individuals come into physical contact with one another. The mobile devices of the users keep track of contact tracing along with the symptoms and infected people. The information is updated on the app server periodically, and the analysis on the data is performed while maintaining the privacy of user identities.

### Safe2

This app uses the integration of both Bluetooth technology and GPS for efficient contact tracing. Bluetooth is utilized to detect droplets of coughs and sneezes, while GPS is used to trace the surfaces in contact with the infected individuals. Self-assessment results are stored and never leave the user’s device until a positive symptom is detected [17]. When the user gets infected, all the data (anonymized close contacts, location history, and exposure status) are uploaded to the alerting system of the app, which keeps user identity anonymous and notifies other users who have been in close proximity with the infected person. The Safe2 app managers have the right to release the user’s personal data to the government or law enforcement agencies if required.

### Aarogya Setu App

This app was developed by the Government of India for the purpose of COVID-19 contact tracing. However, it remained controversial for a long time because people were worried about their privacy. This forced the Indian government to come out with a privacy policy for the app, elaborating on the type of data that is collected and how the data are used. Prominent features include automatic contact tracing via Bluetooth, self-assessment tests, travel advisories, risk status, location data, nationwide COVID-19 status, emergency helpline numbers, and more [18,19]. When an Aarogya Setu app user comes within the Bluetooth proximity of another user, the two devices securely exchange digital signatures related to this interaction. It contains the location data, time, proximity, and duration. These data are stored on the devices of all individuals. When a person tests positive, an alert notification is sent to all users who have been in proximity to that individual within the last 14 days and recommends suitable action. The updated risk of infection is closely monitored by the Government of India.

### Exposure Notification

**Figure 2:**
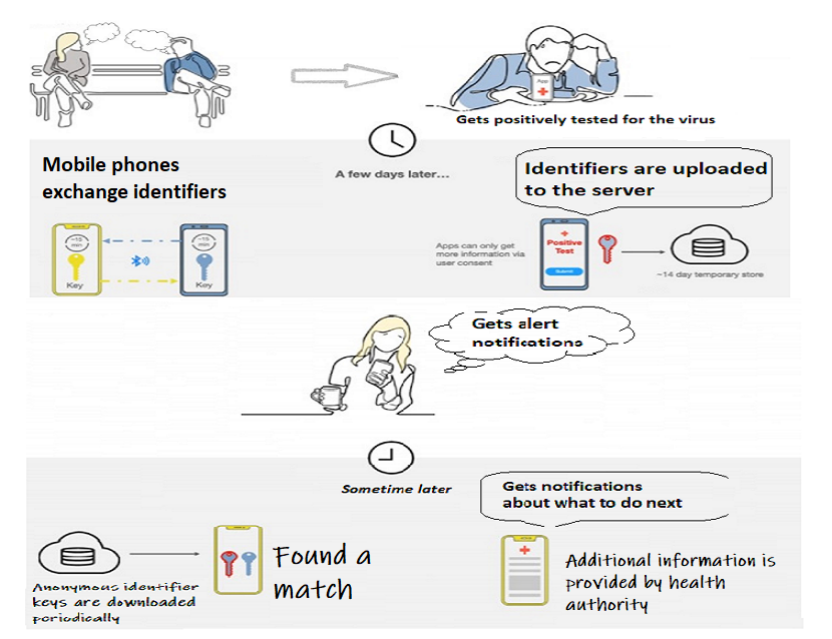
The Exposure Notification system.

Google and Apple have recently announced a joint effort to fight COVID-19 by using Bluetooth technology for contact tracing to help governments and health care organizations to contain the spread of this virus. Called Exposure Notification, this system has been designed keeping in mind the importance of user privacy and security. A random ID is generated when a user installs the Exposure Notifications system on their mobile phone. To preserve the user’s privacy, these random IDs change every 10-20 minutes so that the identity of the user or the geographical location cannot be detected. The user’s mobile phone and those nearby will then continue to exchange these random IDs via Bluetooth. Data are collected, stored, and processed on the user’s mobile phone only. If, at any point in time, a user is diagnosed as positive for the virus, he or she updates their status in the app. Other users’ devices concurrently and occasionally match all the random IDs with positive COVID-19 cases against its own random IDs. During the whole process, the user’s identity is not shared with anyone—not even with Google and Apple. Figure 2 outlines how the Exposure Notification system works [20].

Users’ privacy is ensured as follows:

- Users have full control over the Exposure Notification system. It is up to the user when to turn the app on or off;
- The geographical location of the user is not shared with anyone—not even with the government, Google, or Apple;
- To preserve user privacy, the random Bluetooth IDs change every 10-20 minutes;
- Notifications are carried out on the user’s mobile phone only. To preserve the privacy of the user, if a person tests positive, their identity is not shared with other users or with Apple or Google;
- The objective of this system is to help the health care system fight COVID-19, which is why Google and Apple expect to deactivate this system when it is no longer needed.

**Table 1:** Existing contact tracing techniques for COVID-19.

App name	Information-gathering technique	Type of data collected	Data storage and management
TraceTogether [10,21]	Bluetooth	Anonymous IDs, proximity of contacts	Data corresponding to anonymous IDs are generated from mobile devices and stored on a central server; the real identity of the users is hidden from other users but known to the server
COVID Trace [11]	Bluetooth	Geographic location, temporal details, self-assessed symptoms	Location and temporal details are stored on the users’ personal device; data used only with permission from users
HowWeFeel [12]	Manual	Age, sex, postal code, self-assessed symptoms	Self-reported data are integrated and stored along with user IDs; storage and management via a central app server
NOVID [13]	Bluetooth and ultrasonic sound waves	Device details, OS version, time, language, Bluetooth, sonic signals specifications, proximity of contacts	Generates user ID and corresponding password; utilization and management by a central server
COVID Shield [14,22]	Bluetooth	Unique random ID, app logs, temporary keys for exposure	Data are not directly uploaded to a server; the generated user ID is uploaded to the central server in a secure manner
Zero (Safemap) [9]	Manual and automatic	Mobile number, email ID, GPS information, IP^a^ address	Data are stored on users’ individual server, managed by a central server
ShareTrace [16]	Bluetooth	Proximity of contacts, symptoms of users, diagnosis results	Data are stored on users’ individual server, managed by a central server
Safe2 [17]	Bluetooth and GPS	Proximity of contacts, locations, self-assessed symptoms, lab test results	Generated random user IDs and data are stored on user devices; federated servers are used for handling data

^a^IP: Internet Protocol.

There are several other apps and protocols that have been developed as a solution to handle the COVID-19 pandemic via contact tracing, such as NHS COVID-19, HealthCodeSystem, Pan-European Privacy-Preserving Proximity Tracing [23], Decentralized Privacy-Preserving Proximity Tracing apps, and others [24]. These apps work in a similar manner to the above-mentioned ones, with some minute differences. However, all of these apps face some issues related to security and privacy of the user’s data. Most have their own central server that stores and manages data, which creates vulnerabilities such as data theft, data manipulation, data leakage, single point of failure, etc. Moreover, there is no verification mechanism provided by these apps that assures whether a particular individual is actually infected or not, as there are chances that a malicious user might put fake information on the app to create panic among other users, which raises issues related to the trust and reliability of the app. Furthermore, the personal data being collected by the apps still remain the main concern of app users. Apart from these apps, there are certain other apps that demand permission to access users’ mobile phone contacts, media, location, files, phone ID, etc. Since these apps are constantly gathering user information for contact tracing, the privacy and security of the user’s data continues to be the biggest concern and needs to be addressed [25].

## Issues and Challenges Posed by Digital Contact Tracing

Contact tracing accelerates the process of identifying the individuals who could have come into contact with infected cases. However, there are certain issues, challenges, and concerns associated with this mechanism that need to be addressed.

### Data Privacy and Compliance With the General Data Protection Regulation

One of the biggest concerns is that the people are hesitant in sharing their information, as they are not sure how their data will be used, who is going to be in charge of their data, and for how long. Contact tracing apps must gather data in compliance with the General Data Protection Regulation (GDPR), which states that any information related to a person that could make him or her directly or indirectly recognizable is said to be his or her personal information. Some important terms related to the GDPR are discussed below [26-29]:

- Data subject: refers to any individual who can possibly be identified directly or indirectly by means of an identifier such as a name, location data, personal ID number, or through some specific factors related to an individual’s physical, genetic, economic, cultural, or social identity;
- Personal data: according to the GDPR, data become personal data when any kind of identification of the subject is possible through that data;
- Data controllers: refers to the main policy and decision makers who control the purpose behind the data collection and method of data processing;
- Data processors: work according to the instructions provided by data controllers to process the data.

In addition, there are various rights given by the GDPR to users regarding data and privacy, which include [30]:

- Right to access: the individual has the right to access personal data. Additionally, they have the right to know how their data are being used, processed, stored, and transferred to other organizations;
- Right to be informed: the individual has the right to be informed before data are gathered and processed;
- Right to data portability: the individual has the right to transfer their data from one service provider to another at any time;
- Right to be forgotten: the individual has the right to have their data deleted if they are no longer customers, or withdraw their consent of data usage;
- Right to object: the individual has the right to object the use or processing of their data;
- Right to restrict processing: the individual can ask to stop processing of certain kinds of data;
- Right to be notified: the individual has the right to be notified within 72 hours in case of a breach of their personal data;
- Right to rectification: the individual has the right to request the data controller to update, modify, or correct their data.

In terms of health care data, the GDPR requires more protection; in the context of digital contact tracing apps, the wireless technology and app managers receive the data along with user identifiers, making it their responsibility to keep the data anonymous. All the strategies for contact tracing available today employ one simple and common mechanism that tracks the movement of the carrier of the virus and does not protect the privacy of users. Therefore, the people who have been diagnosed with the disease are afraid of losing confidentiality since details about their location is broadcasted publicly. Although the actual identity of the individual is not disclosed, they can be mapped easily due to the limited number of carriers on the same route. Once identified, people can start making speculations about their personal lives, develop incorrect perceptions, or generate rumors.

### Data Quality and Transparency

COVID-19 is generating considerable amounts of data such as information on infected people, hospitalization and death rates, transmission details, etc. However, it is necessary to analyze these data so as to gain insights and make better and efficient decisions since the fight against the spread of COVID-19 is dependent on these decisions and the research being carried out. Therefore, it is essential that the data being analyzed are accurate and the quality of the data is assured. It must consist of metadata and context to avoid any misjudgment or discrepancy since lives are at stake. Moreover, the transparency of the data is another critical aspect; the origin and the generator of the data must be known to the researchers and/or users in order to increase trust and prevent the spread of false information or panic.

### Lack of Medical Understanding

COVID-19 is a highly communicable disease that is not only caused by physical contact but can also spread through air, which makes tracking difficult since an individual can become infected without even coming into contact with an infected person [31]. Another issue that hurdles the implementation of contact tracing is that scientific data on COVID-19 are limited at present. For example, it affects each individual differently. Some patients are facing difficulties in breathing and dying while others experience no symptoms but are found to be COVID-19 positive. Additionally, asymptomatic and presymptomatic patients can also infect other people, which makes it difficult to track the source of contamination, as there is a chance that person A, who was asymptomatic and unaware of their infection, came into contact with person B, who became infected and experienced a severe outcome (eg, complications or death) because of the infection.

### Availability of Testing

The accuracy and efficiency of contact tracing is completely dependent on the amount of testing being performed. As of now, the government is only conducting tests on those who have a history of contact with an infected person or is experiencing symptoms related to COVID-19. Moreover, fees for the diagnostic tests are very high, making it difficult for the public in poor countries to get tested on their own.

### Trust Issues Between Governments and Citizens

Several contact tracing apps have been developed in the past few months by governments to combat the spread of the COVID-19 pandemic; however, many people criticize this as they believe this to be an attempt by their governments to regulate their lives and breach their fundamental rights of privacy. For example, Israeli legislation has allowed government officials to monitor the mobile phone data of people suspected of infection [32]. Similarly, a publicly available database has been created by the government of South Korea that consists of the personal information of infected individuals such as their job, travel routes, gender, age, etc [33]. Furthermore, GPS-based apps capture and broadcast the location details of users, which invades individuals’ privacy [34]. Thus, it is necessary that the information being shared by users must be handled lawfully and with user consent.

### Technical Inefficiencies

Various digital contact tracing techniques based on GPS tracking and Bluetooth technology have been employed in the past. However, there are certain issues with these technologies relating to surveillance, malicious users, snoopers, etc. One such technique based on Bluetooth was proposed in 2018 [35], which was used to detect whether or not certain individuals came into proximity with infected people. This can be estimated on the basis of the strength of Bluetooth signals, which becomes low if there are any kinds of obstacles in between (like walls). Thus, considering the current scenario where there is an abundance of large buildings, the effectiveness of such a technical solution is highly affected. Moreover, the adoption rate of such systems is not very high, which is also a reason behind its limited effectiveness. Although GPS has been used to retrieve the location details of users in a digital contact tracing app, it is not very safe and efficient since it is very easy for malicious attackers to create false information on GPS networks [36]. In addition, there is a chance that an app might provide users with false notions of safety. It is possible that a person might have come into contact with an infected person but failed to be notified by the app, thus the user remains unaware of the exposure and does not follow preventive measures.

## Probable Attacks on Digital Contact Tracing Apps

**Figure 3:**
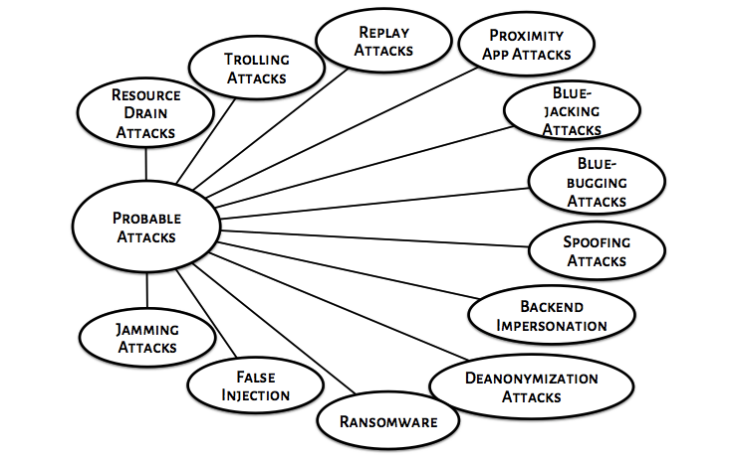
Probable attacks on contact tracing apps.

Figure 3 displays some of the most common ways digital contact tracing apps can be compromised by a malicious user/attacker. Some of these attacks are generic while others are specific to the wireless technology being used [37].

### Resource Drain Attacks

This is one of the most common types of attack that leads to denial of service. In such attacks, the attacker sends a massive number of trash messages, either valid or invalid, from their device to the server that forces the drainage of the other device’s resources (eg, battery life) [38]. These attacks have no impact on the digital contact tracing app but might lead to poor performance of the mobile device.

### Trolling Attacks

In a trolling attack of a digital contact tracing app, the attacker spreads false information, such as lying about being exposed to the virus or being in close proximity with someone who has been diagnosed with the virus [34]. This misinformation creates panic among users, leading them to conduct diagnostic tests and inducing loss of trust in the system.

### Replay Attacks

In replay attacks, the attacker uses one or more mobile device(s) at different instances to broadcast the same message multiple times. In the context of digital contact tracing apps, replay attacks might spread anxiety among the public as a malicious user who is diagnosed positive with the virus pretends to be more than one individual and sends alerts to even those users who were not near them [39].

### Proximity App Attacks

The proximity information of the user is supposed to be confidential, but an attacker can hack into a user’s device and leak the information on users’ daily life events to others. This attack creates fear among users and makes them reluctant in using the app and sharing their proximity details.

### Blue-Jacking Attacks

These attacks are specific to Bluetooth-based digital contact tracing apps, in which the attacker exploits Bluetooth and sends spam messages (containing the name and model of the sender device) to other devices that have their Bluetooth turned on. The receiving user has no idea about the sending user. The aim of such attacks is to make the receiving user behave in a certain manner and access the receiver’s device.

### Blue-Bugging Attacks

Blue-bugging attacks are also specific to Bluetooth technology. In such attacks, the attacker tries to gain unauthorized access to a user’s device and attempts to give commands. It is the most dangerous attack, as the attacker might acquire total control over the user’s device and misuse personal information.

### Spoofing Attacks

These attacks are usually common in digital contact tracing systems based on GPS technology, where the attacker exploits GPS signals near a mobile device in order to transmit incorrect location data that affects the overall performance of the tracing apps and results in incorrect judgments and decisions [40].

### Backend Impersonation Attacks

In such attacks, the attacker pretends to be someone else by replacing their own identity with the identity of another device. Then false information is broadcasted to other devices within the network.

### Deanonymization Attacks

Deanonymization attacks disrupt the privacy of users. The main focus of such attacks is the disclosure of the identity of the user to the entire network, which could lead to social stigma or discrimination if the user’s diagnosis is found to be positive.

### Ransomware

In ransomware, the attacker develops fake contact tracing apps to gain access to users’ mobile phones. This type of attack misuses the pandemic situation to gain information and data of users through their phones such as bank details, photos, contacts, etc [41]. Moreover, the attacker might lock the phone and can demand money to unlock it.

### False Injection Attacks

In false injection attacks, the attacker introduces deceptive information and tries to compromise information transactions among users, which lead to poor performance in tracing apps. If the attacker becomes capable of getting control over multiple devices within the network, the attacker may be able to create fraudulent reports.

### Jamming Attacks

Jamming attacks are specific to GPS-based contact tracing apps, in which signal interference (intentional or unintentional) prevents genuine signals from being received by users. The main objective of such attacks is to disrupt tracking via GPS signals, hence affecting the overall performance of the contact tracing system.

## Blockchain-Based Digital Contact Tracing for COVID-19

Digital contact tracing produces certain issues and challenges related to the privacy and security of users’ personal information as discussed in the sections above. Since users’ information is collected, traced, tallied, and broadcasted to the network, it becomes necessary to maintain confidentiality and prevent the divulgement of the user’s identity. However, in certain apps, consent is asked of users, which provides some control to users over their information. However, verification of the authenticity of the information being shared is still lacking, as well as assurances to the users that only data relevant for tracing COVID-19 spread is being captured and nothing else. Blockchain technology can play a significant role in contact tracing as it supports the distributed peer-to-peer connectivity of the network nodes that bridges the gap between the users and app managers. The technical features offered by blockchain allow for sharing of information while preserving the privacy of users [42]. Table 2 provides a brief description of how the default properties provided by blockchain technology can be utilized for contact tracing apps.

**Table 2:** Features of blockchain technology and the corresponding application in the digital contact tracing process.

Feature	Application
Decentralized network	The management of the data is user centric, which gives the power of data ownership to the users
Data security	The data within the blockchain is kept after applying encryption, which can only be decrypted by an authorized user
Data provenance	The information being entered in the blockchain is stamped with the digital signature of the source, which proves the legitimacy of the source as well as the data
Data availability	The data are distributed among all the nodes within the network, which makes them available all the time to every user
Data immutability	The information in the blockchain is immutable, which means once a detail is entered it can never be modified. This provides reliability and transparency to all users
Time stamping	The data within the blockchain network is time stamped, which eliminates the chances of discrepancies being present

The privacy provided to the user and the performance of the app in terms of tracing contacts effectively should be the main focus of these apps. As most of the available apps lack the capability to protect user privacy, the main concern today is to provide effective tracing without compromising the users’ identity and privacy [43,44]. Furthermore, the sharing of the data for decision-making processes in a secure manner is another challenge that is difficult in a centralized network due to the risks of data manipulation. This could be handled in blockchain-based systems as the network is totally distributed and the identities of the users are made anonymous in the beginning. Apart from preserving users’ privacy, the app should also perform effectively in terms of tracing possible contacts, network coverage, infection prevention, etc. Currently, the apps that support decentralization are available for small geographical networks, which would not be beneficial for those who travel on a daily basis for work. Therefore, a blockchain-based decentralized network can provide accessibility and traceability at a global level and connect a larger number of users from different geographical locations without compromising their privacy. Moreover, the information shared on blockchain can be collected from any means of technology (Bluetooth, GPS, etc) and can provide better and richer interactions.

**Figure 4:**
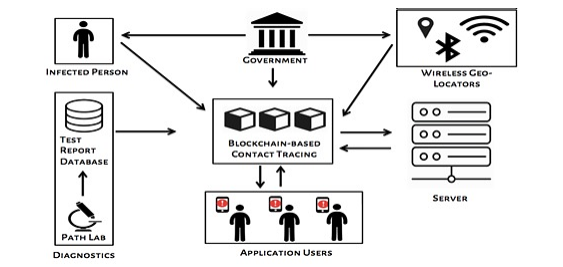
Blockchain-based contact tracing app framework.

**Figure 5:**
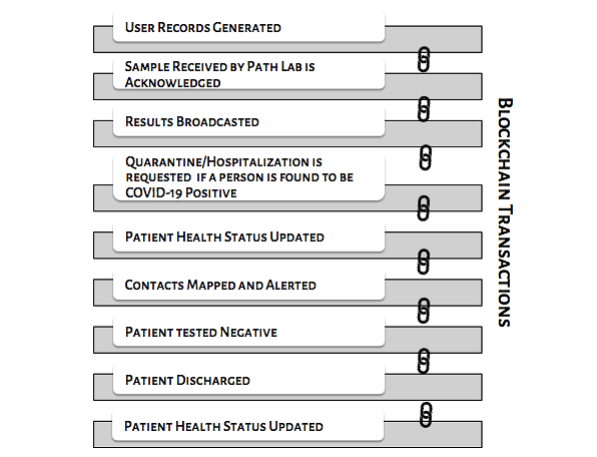
Data transaction flow in a blockchain-based contact tracing app.

The spreading of false information and rumors that might lead to panic among people is very dangerous and should be prevented. The reason behind the false information could be attributed to inaccurate details or a lack of transparency. It is necessary for health care organizations to have trustworthy authorities to verify and validate the information received to avoid inaccuracy or discrepancy in the data. Therefore, the blockchain network would be the best option, as it provides transparent tracing of contacts while maintaining privacy in a verifiable manner. Moreover, the privacy of the data should be ensured for the entire lifecycle of the data from its generation to its disposal, which is usually 14 days in the case of COVID-19. Blockchain technology provides users with full control over the management of their data from start to finish and allows them to share and withdraw their data any time they want. Figure 4 depicts a typical blockchain-based contact tracing app framework that shows how the information regarding proximity and health status of the user are collected, analyzed, and utilized. Any activity within a blockchain-supported architecture takes place in the form of transactions, and these transactions are requested and represented in the form of blocks. These blocks of transactions are broadcasted to every node of the network and validated only if the nodes within the network verify them. Figure 5 illustrates the flow of possible transactions in a blockchain-based contact tracing app.

All users of the app, including the infected ones, will upload their data, that is, their unique ID and geographical information after applying encryption, to the blockchain network and perform matching on their own devices. When a user goes to the diagnostic center for a test, the results would be uploaded to the blockchain contact tracing app, which would then map the infected person to retrieve the details of their contacts with the help of the server. The servers basically read the data from the blockchain, and the network does the mapping using the geographical data available and provides the results back to the blockchain. The geographical data provided to the servers are collected using wireless technologies, such as Wi-Fi, Bluetooth, or GPS, that capture the location details of the users. The government monitors all data transactions occurring between users, path labs, server, geo-locators, and the blockchain.

## The GDPR and Privacy Preservation in Contact Tracing Apps

Privacy experts fear that the widespread use of contact tracing apps could disrupt the development of privacy-preserving regulations. In this context, some of the concerns addressed by the GDPR include [45]:

- Emphasis is on Bluetooth low-energy technology rather than the collection of location data from users through contact tracing apps;
- When it comes to using location data, preference should always be given to the processing of anonymized data rather than personal data;
- Once the user is diagnosed as infected with the virus, only the persons with whom the user has been in immediate proximity and within a specified time period should be informed;
- Information related to proximity between users of contact tracing apps can be obtained without locating them. The main motive is that the apps should not use the location data;
- Given the complexity of anonymization processes, transparency regarding the anonymization methodology is highly encouraged;
- If the app is based on a centralized type of architecture, then in accordance with the ethics of data minimization and data protection by design, the data processing capability of a centralized server should be restricted to minimum;
- Information kept on a central server should not allow the controller to recognize infected users or those people who were in proximity of an infected person. Additionally, it should not allow for the interpretation of contact patterns that could lead to the identification of contacts;
- To preserve privacy, it is adequate to exchange pseudonymous identifiers through Bluetooth low-energy technology across the users’ device (mobile phone, tablet, watches, etc);
- These pseudonymous identifiers must be generated using various state-of-the-art cryptographic processes;
- The identifiers must be renewed at regular intervals of time to reduce the risk of physical tracking and linkage attacks;
- The app should not convey to the users information that allows them to infer the identity or the diagnosis of others. The central server must neither identify users, nor infer information about them.

## Discussion

The prevention of subsequent waves of COVID-19 transmission is the top priority in many countries around the world. Consequently, contact tracing is gaining much attention. Contact tracing is a highly effective cornerstone in the fight against infectious diseases. A similar version can be used to identify various other infectious diseases, but this cannot be generalized to COVID-19. Alternatively, COVID-19 tracing involves observation or surveillance to detect the early outbreak of this infectious disease, protect the population, and monitor testing resources properly [46].

Other issues include:

- Testing resources need to be enhanced. In addition, the various protocols and guidelines for state-level testing need to be evaluated;
- Research suggests that contact tracing has been most effective in places where the number of circulating cases is low;
- COVID-19 essentially has both a long period of infectiousness spread and asymptomatic spread. This makes manual contact tracing difficult because its success relies on an individual’s ability to recall contacts while having been contagious, including prior to feeling ill;
- Research suggests that contact tracing and associated quarantine policies are not 100% effective, which burdens individuals in underresourced communities.

These challenges are real, but contact tracing needs to be done regardless because we have yet to come up with an effective treatment for COVID-19 [47]. Despite all these challenges, we need to adjust our strategies for handling this virus. Possible measure can be:

- Secure and confidential public health surveillance systems that are integrated with standardized reporting infrastructure;
- Monitoring of health system reports of patients feeling unwell and observe for concerning spikes, if any;
- Implementation of an enhanced surveillance model;
- Direct outreach to underresourced communities to monitor their health conditions to help expand the efforts against COVID-19;
- Prioritizing testing in high-risk groups to maximize the benefits of testing and consequent contact tracing.

Recently, a few entrepreneurs from India devised clever artificial intelligence (AI)–enabled technologies to keep workers safe from COVID-19. In a factory, for example, as soon as someone becomes infected, transmission is quick. As a result, it can be difficult to plan for production in such a work setting when it is unknown who is coming to work, who is well, and who is unwell. In order to curb this, one organization named BLP Group devised three AI solutions involving contact tracing to handle these challenges:

- Smart cameras monitored workers and detected whether or not they were wearing a mask via a model that uses computer vision to scan the workspace. This allowed for the detection of a person who was in close proximity to someone else as well as if they were wearing a mask. The cameras could even carry out regular temperature checks using thermal imaging. The issue is that employees must feel comfortable with these technologies. The right communication was critical to relay to workers the purpose of the tool (safety rather than policing). As soon as the workers were able to understand this purpose in terms of its benefits, they started responding to it well;
- Mobile phones were turned into an automatic alert system. When workers got too close to one another, their mobile phones started vibrating and ringing so as to create a warning system with no need for cameras;
- Wearable devices in the form of a wrist band buzzed when social distancing was breached.

Furthermore, if a worker became sick, data were collected from their wearables and contact tracing was quickly employed. This will, in turn, enable businesses to operate and isolate people who fall in the risk group. These technologies are also being used in some airports, hotels, and offices. This could be a way of building safer workplaces even when the pandemic is over. Such types of strategies can transform and disrupt the way businesses are carried out and could be used to formulate strategies for combating COVID-19.

## Conclusion and Future Directions

The novel coronavirus has affected more than 42 million people throughout the globe and has been termed a global emergency by the WHO. The virus is highly contagious and spreads through physical contact and socializing; therefore, in order to combat such diseases, several digital contact tracing mechanisms have been developed by companies, researchers, and governments. Contact tracing techniques have been practiced for many years now, but with time they have evolved and now utilize wireless technologies and mobile devices. Nevertheless, users are reluctant in sharing and broadcasting their personal information and proximity details, due to certainties about how their information will be used, by whom, and for how long. Therefore, a blockchain-based digital contact tracing technique that efficiently provides contact tracing without compromising users’ privacy or confidentiality is required. Blockchain provides users with the total control over their data throughout the lifecycle and allows for withdrawal at any time. Moreover, the data stored are encrypted, time stamped, and immutable, making access by unauthorized persons impossible, which promotes transparency and eliminates discrepancy.

The success of any digital contact tracing app is dependent on trust and reliability and affects performance and wide-scale user adoption rate. In addition, the coverage of digital contact tracing apps should be high, as the apps can only run on smartphone devices, which makes its adoption in some countries like India, Bangladesh, and Pakistan, etc, challenging, as the marketing and usage of smartphones in such countries is low. People who are uncomfortable with technology may not want to run these apps, leading to unawareness of their proximity and exposure. Bluetooth is the most widely used wireless technology in digital contact tracing apps and its proximity range is relatively high. However, it is not intelligent enough to detect any object in between 2 devices such as walls or doors. It might create problems in cities where homes are congested and generate incorrect results such as false positives that might lead to panic among people [15]. To handle this, Apple-Google API has included the transmission signal strength, but this area requires more research.

Another domain that needs work in these apps is the promotion of trust between citizens (users) and governments. As the proximity of users is being broadcasted at all times with these apps along with their personal data, users can become suspicious about being surveilled by their government. Therefore, the information being shared by users must be handled lawfully, with user consent, and in compliance with the GDPR to keep personal information private and identities anonymous [48,49]. Some countries like China and India have made their contact tracing app mandatory for every citizen, while others have made it voluntary whereby only interested users can download and use it. Since the disease is spreading at a faster rate, it becomes necessary to have trust among citizens to improve adoption rates. Moreover, these apps should be transparent and ask for consent to assure users in terms of security and privacy. Apart from the aforementioned concerns, other issues needing improvement that affects app performance and user acceptance rates include glitches in the apps, interoperability, and proper detection of devices [50].

## Abbreviations

AI: artificial intelligence
API: application programming interface
AWS: Amazon Web Services
GDPR: General Data Protection Regulation
SSL: Secure Socket Layer
TCN: Temporary Contact Numbers
WHO: World Health Organization
